# Supplementary figures and images for: A broad analysis of splicing regulation in yeast using a large library of synthetic introns
Source: PLoS Genet. 2021 Sep 27;17(9):e1009805. doi: 10.1371/journal.pgen.1009805 (PMC8496845; doi:10.1371/journal.pgen.1009805)

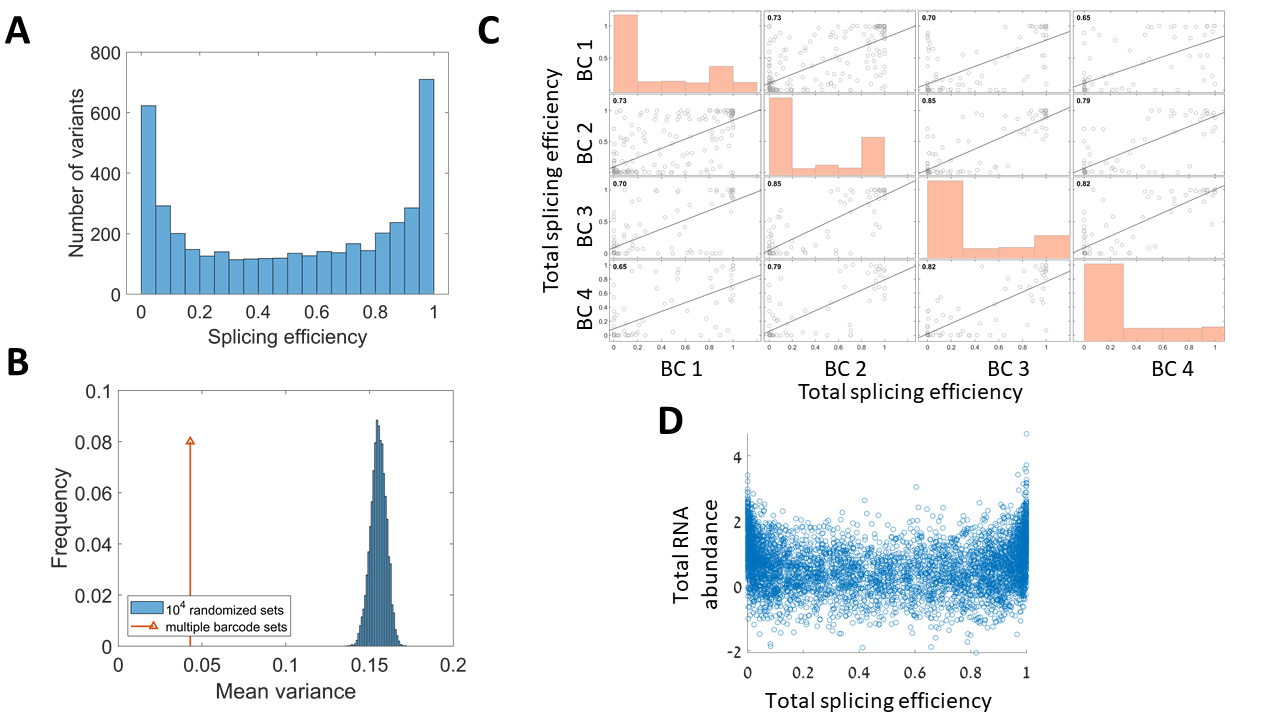

Supplement: S1 Fig — A. Histogram of total splicing efficiency values. Only variants with splicing efficiency > 0 are presented. B. Splicing efficiency mean variance of quartets of the same sequence design, with different barcode sequences (red arrow), compared to the distribution of mean variance of 10,000 sets of quartets, randomly chosen from the set of designs with multiple barcodes. C. Pairwise Pearson correlations of total splicing efficiency values (i.e. splicing efficiency of intended + cryptic spliced isoforms) between designs with identical sequence and different 12nt barcode sequence (mean Person correlation, r = 0.76). D. To check if the correlation between RNA abundance and splicing efficiency results trivially from the dependence of splicing efficiency value on the total RNA abundance, we ran the same analysis as in Fig 3A on a randomized dataset. For 5,000 mock variants we randomly assign unspliced RNA levels, and spliced RNA levels. Both values are randomly chosen from a log-normal distribution. We then calculate the splicing efficiency of each mock variant, and plot the scatter of total RNA abundance and splicing efficiency. No significant correlation is observed in this mock data (p-value = 0.45). (TIF) [file pgen.1009805.s001.tif]

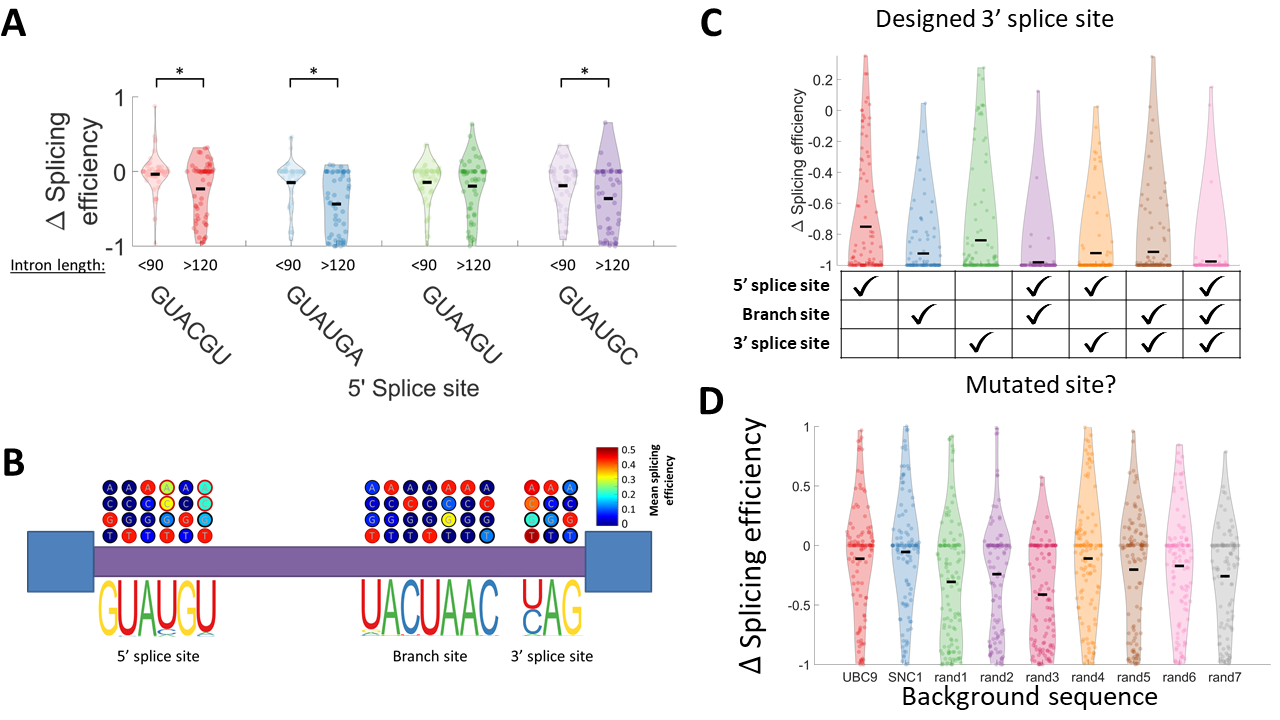

Supplement: S2 Fig — A. For two of the 5’SS variants (i.e. GUACGU, GUAUGA), the difference in splicing efficiency as described in Fig 3D, is significantly lower only for introns longer than 120 nucleotides. This figure presents the distribution of the difference in splicing efficiency for 5’SS variants after binning variants according to their intron length. B. Single mutation analysis of splice sites’ positions. For each site, the bottom part presents the sequence logo of the consensus splice site, and above it a depiction of the effect of each mutation on the proportion of spliced variants. For each mutation, the other positions within splice sites are kept at the consensus sequence. Color bar represents the mean splicing efficiency of all variants with this mutation as a single mutation in the splice sites. Values significantly higher than zero are marked with a red circle (proportion z-test, with Bonferroni correction). C. Distribution of difference in splicing efficiency for variants with mutations near splice sites which result in predicted secondary structure in which the splice resides within the stem of a stem loop structure. The differences are taken compared to an unmutated variant with the same splice site sequences and length properties. For all possible combination of splice site structure mutations, the distribution of splicing efficiency is significantly lower than the splicing efficiency of the reference variant (t-test, p-value<10−40). √ symbols mark the mutated sites. D. Distribution of differences in splicing efficiency for different background sequences, compared to a reference variant with the same design features and the MUD1 background sequence. Splicing efficiency distribution for the SNC1 background is not significantly different from MUD1 distribution (t-test, p-value = 0.2), other background sequences have significantly lower distribution (t-test, p-value<0.01). (TIF) [file pgen.1009805.s002.tif]

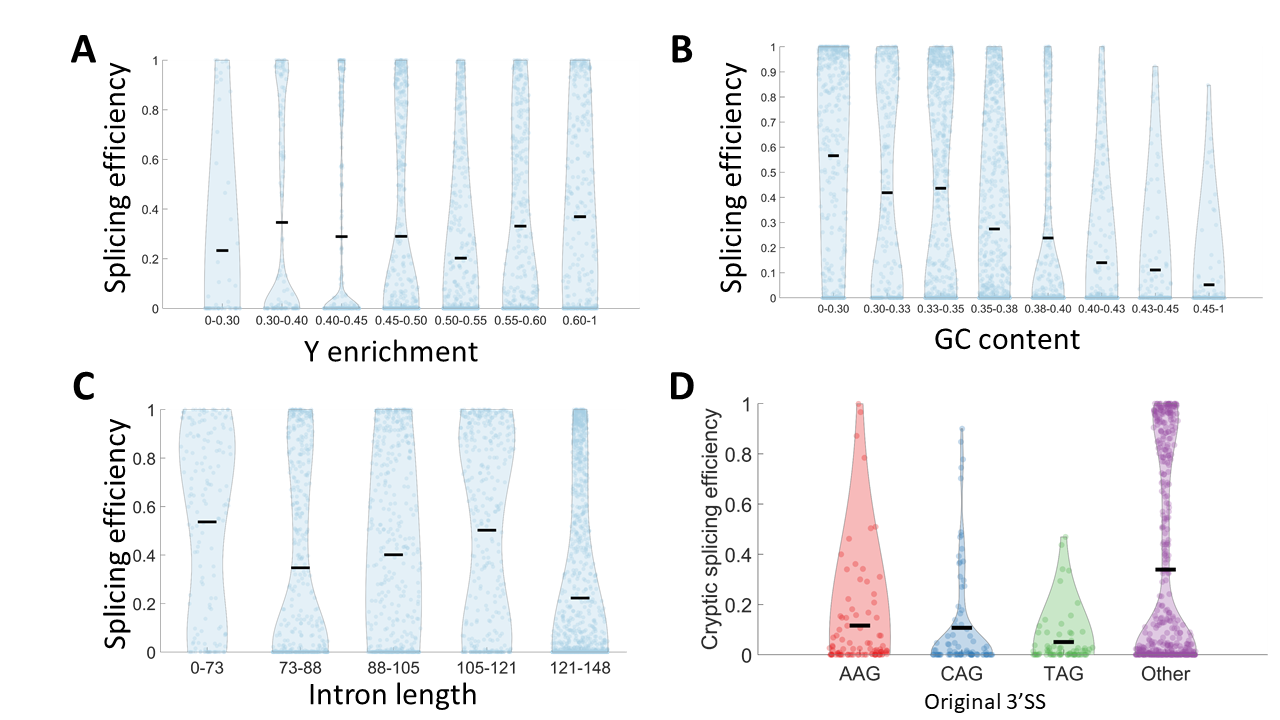

Supplement: S3 Fig — A-C. These panels of this figure present a comparison of splicing efficiency values for all the library variants utilizing consensus splice sites’ sequences. A. Splicing efficiency distribution is binned according to poly-pyrimidine tract strength, which is calculated as the Y (i.e. C or U) content at a window of 20 nucleotides upstream to the 3’SS. In order to specifically check elements that are not U-rich, only elements with at least 30% C out of their Y content are taken into account. Correlation is not significant (p-value = 0.79), compared to the highly significant correlation for U-rich elements (Fig 4A). B. Splicing efficiency distribution binned according to intronic GC content (Pearson r = -0.85 p-value<0.01). C. Splicing efficiency distribution of spliced variants for different intron lengths. D. Distribution of cryptic isoforms splicing efficiency for the set of synthetic introns, binned according to the original intron’s 3’SS. (TIF) [file pgen.1009805.s003.tif]

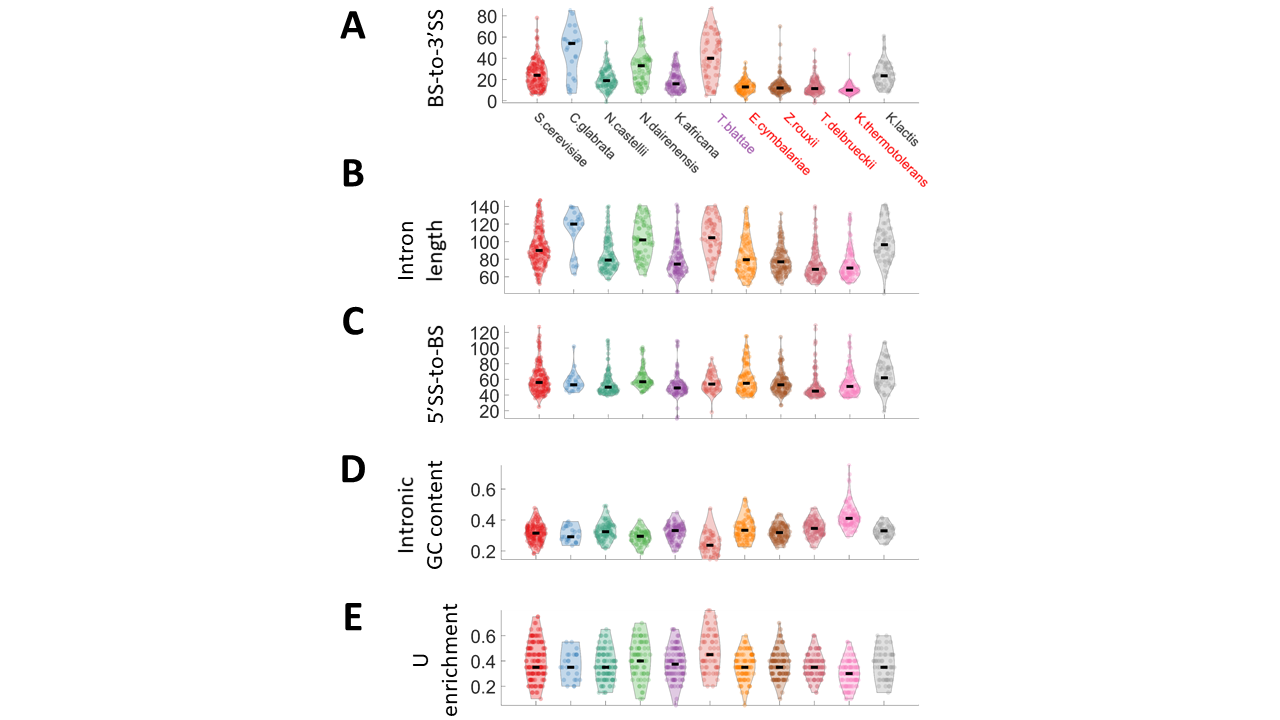

Supplement: S4 Fig — A. Distribution of BS-to-3’SS distance in each of the 11 species. Species with no copy of the gene coding for the splicing factor U2AF1 are marked in black, one species with a malfunctioned copy of U2AF1 is marked in purple, and the ones with a functional copy of U2AF1 are marked in red. B-E. For the same species represented in (A), and in corresponding positions, distribution of intron length (B), 5’SS-to-BS distance (C), intronic GC content (D), and poly uracil enrichment as calculated in Fig 4A. We notice that although intron length differs substantially between species with U2AF1 splicing factor, to species that lack it (B), this difference is ascribed solely to differences in BS-to-3’SS distance (A), as we see no difference in 5’SS-to-BS distance (C). (TIF) [file pgen.1009805.s004.tif]

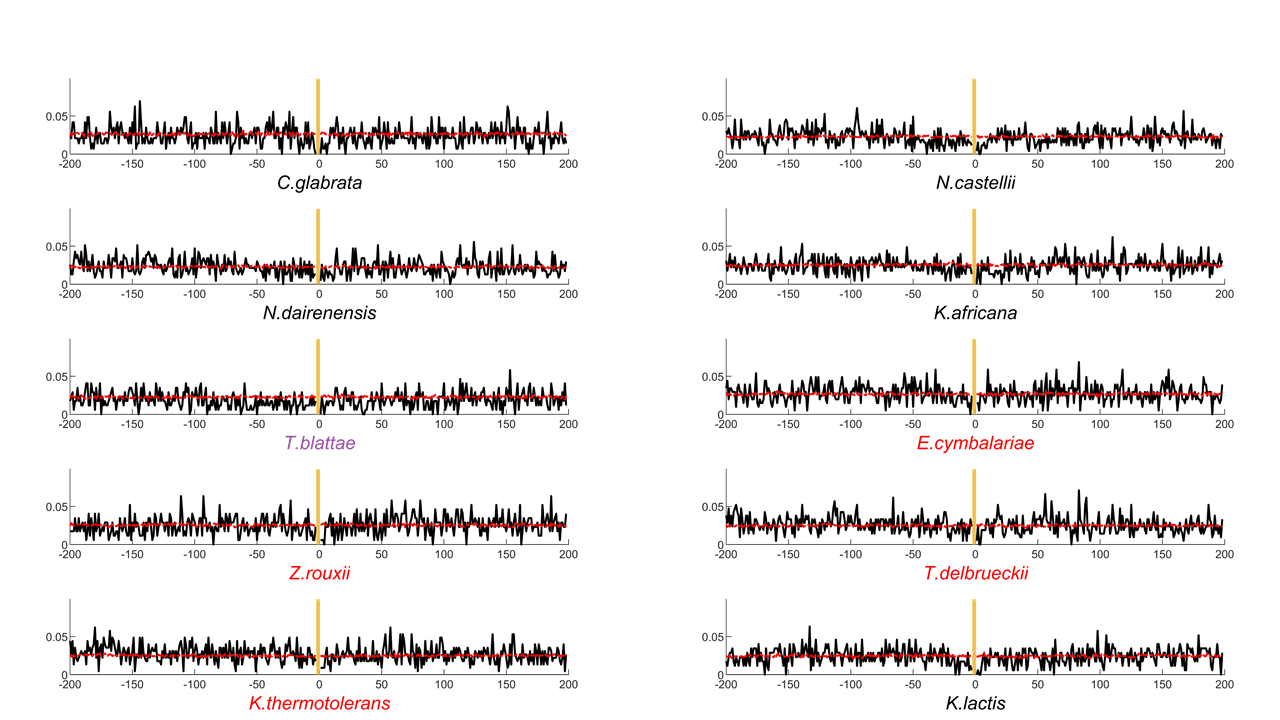

Supplement: S5 Fig — 3’ splice site motif avoidance signal for each of the other 10 yeast species that contributed introns to the library. Each panel presents the motif avoidance signal as explained in Fig 5F. (TIF) [file pgen.1009805.s005.tif]
