# Supplementary material for: A broad analysis of splicing regulation in yeast using a large library of synthetic introns
Source: PLoS Genet. 2021 Sep 27;17(9):e1009805. doi: 10.1371/journal.pgen.1009805 (PMC8496845; doi:10.1371/journal.pgen.1009805)
Supplement: S1 Table — (PDF) [file pgen.1009805.s006.pdf]

Table S1 - Synthetic combinatorial design library subset features

Feature name	Possible values
5’SS sequence	'GUAUGU'; 'GUACGU'; 'GUAUGA' ;'GUAAGU'; 'GUAUGC'
Negative control 5’SS sequence	'AUUGUG'; 'CGAUGG'
BS sequences*	'UACUAAC'; 'NNCUAAC’; ‘NNCUAAU’; ‘NNUUAAC'
Negative control BS sequences	'CAUAUCA'; 'AUCGAGC'
3’SS sequences	'UAG'; 'CAG'; 'AAG'
Negative control 3’SS sequences	'AGU'; 'CAU'
Intron lengths [Nucleotides]	73, 89, 105, 121, 137
BS-to-3’SS lengths [Nucleotides]	20, 30, 40, 50
3’ U-rich sequence element	‘AUUUUUAA’; ‘UUUAA’; ’UAA’

* - In BS sequence variants since in the genome there is variation in these positions for non-consensus variants, ‘N’ nucleotides were replaced with a randomly chosen nucleotide. For the 'NNCUAAC' variant, only variants different than 'UACUAAC' are considered (as this is the consensus sequence).
