# Supplementary material for: A broad analysis of splicing regulation in yeast using a large library of synthetic introns
Source: PLoS Genet. 2021 Sep 27;17(9):e1009805. doi: 10.1371/journal.pgen.1009805 (PMC8496845; doi:10.1371/journal.pgen.1009805)
Supplement: S2 Table — (PDF) [file pgen.1009805.s007.pdf]

Table S2 - gradient boosting model features

Feature name	Feature type
5’SS sequence	categorical
BS sequence	categorical
3’SS sequence	categorical
Intron GC%	numeric
U-enrichment @ 3’ end	numeric
Intron length	numeric
BS-to-3’SS length	numeric
5’SS ΔG (30nt window)	numeric
BS ΔG (30nt window)	numeric
3’SS ΔG (30nt window)	numeric
3’SS GC% (30nt window)	numeric
5’SS stem length	numeric
BS stem length	numeric
3’SS stem length	numeric
5’SS stem arm	numeric
BS stem arm	numeric
3’SS stem arm	numeric
5’SS fraction of nucleotides based paired	numeric
BS fraction of nucleotides based paired	numeric
3’SS fraction of nucleotides based paired	numeric
5’SS - is 1st nucleotide paired	categorical
5’SS - is 2nd nucleotide paired	categorical
5’SS - is 3rd nucleotide paired	categorical
5’SS - is 4th nucleotide paired	categorical
5’SS - is 5th nucleotide paired	categorical
5’SS - is 6th nucleotide paired	categorical
BS - is 1st nucleotide paired	categorical
BS - is 2nd nucleotide paired	categorical
BS - is 3rd nucleotide paired	categorical
BS - is 4th nucleotide paired	categorical

BS - is 5th nucleotide paired	categorical
BS - is 6th nucleotide paired	categorical
BS - is 7th nucleotide paired	categorical
3’SS - is 1st nucleotide paired	categorical
3’SS - is 2nd nucleotide paired	categorical
3’SS - is 3rd nucleotide paired	categorical
