# Supplementary material for: A broad analysis of splicing regulation in yeast using a large library of synthetic introns
Source: PLoS Genet. 2021 Sep 27;17(9):e1009805. doi: 10.1371/journal.pgen.1009805 (PMC8496845; doi:10.1371/journal.pgen.1009805)
Supplement: S3 Table — (PDF) [file pgen.1009805.s008.pdf]

Table S3 - List of primers

Name	Used for	Sequence
prDS1	NEBuilder assembly of reporter cassette	CTCATAAGCAGCAATCAATTCTATCTATACTTTAAAATGCTTTCTGCATCTATATTACCCTGTTATCCC
prDS6	NEBuilder assembly of reporter cassette	GATCGGCTTACTAATATGGGGCCGTATACTTAC
prDS7	NEBuilder assembly of reporter cassette	ACGGCCCCATATTAGTAAGCCGATCCCATTAC
prDS8	NEBuilder assembly of reporter cassette	TCACCTTTAGACATTTTATGTGATGATTGATTGATTG
prDS9	NEBuilder assembly of reporter cassette	AATCATCACATAAAATGTCTAAAGGTGAAGAATTATTCACTGGTGT
prDS10	NEBuilder assembly of reporter cassette	CTGGTTGAAACAAATCAGTGCCGGTAACGCTTTTTGTATCTTGAGTCGACACTGGATGGCGGC
prDS20	RF cloning pBAR3	CCTTCGTTCTTCCTTCTGTTCGGAGGGGACCAGGTGCCGTAAG
prDS21	RF cloning pBAR3	CCGGGTGACCGATTCGGTAATCCCGGTAGAGGTGTGGTCAATAAG
prDS22	Linearize pDS101	TCCGAACAGAAGGAAGAAC
prDS23	Linearize pDS101	GATTACCGAATCGGTCAC
prDS55	Amplification & cloning SplicingLib1 index1	AAAAGTGGAAGTCAGGGTGTTGGTGTAAAGAACATCTAAATACGAGGCACTTACTCCG
prDS56	Amplification & cloning SplicingLib1 index2	AAAAGTGGAAGTCAGGGTGTTGGTGTAAAGTGTTGGGAAATACGAGGCACTTACTCCG
prDS57	Amplification & cloning SplicingLib1 index3	AAAAGTGGAAGTCAGGGTGTTGGTGTAAAGAAGCCATGAATACGAGGCACTTACTCCG
prDS58	Amplification & cloning SplicingLib1 index4	AAAAGTGGAAGTCAGGGTGTTGGTGTAAAGGCTAAAGAAATACGAGGCACTTACTCCG
prDS59	Amplification & cloning SplicingLib1 R	ATTGTGGGGAGTGGAACGCAGTCACATTGATAGGAATAGCGAACTCCAGG
prDS62	Linearize pDS102	CTTTACACCAACACCCTGAC
prDS63	Linearize pDS102	TCAATGTGACTGCGTTCCAC
prDS137	NGS library preparation shift0	ACGACGCTCTTCCGATCTGTCAGGGTGTTGGTGTAAAG
prDS138	NGS library preparation shift1	ACGACGCTCTTCCGATCTAGTCAGGGTGTTGGTGTAAAG
prDS139	NGS library preparation shift2	ACGACGCTCTTCCGATCTTCGTCAGGGTGTTGGTGTAAAG
prDS140	NGS library preparation shift3	ACGACGCTCTTCCGATCTCATGTCAG

		GGTGTTGGTGTAAAG
prDS141	NGS library preparation shift4	ACGACGCTCTTCCGATCTACTAGTCAGGGTGTTGGTGTAAAG
prDS142	NGS library preparation shift5	ACGACGCTCTTCCGATCTTAGCCGTCAGGGTGTTGGTGTAAAG
prDS143	NGS library preparation R	AGACGTGTGCTCTTCCGATCTGTGGAACGCAGTCACATTGA
prDS144	NGS library preparation PCR2	AATGATACGGCGACCACCGAGATCTACACTCTTTCCCTACACGACGCTCTTCCGATCT
prDS145	NGS library preparation PCR2 with index	CAAGCAGAAGACGGCATACGAGAT[index]GTGACTGGAGTTCAGACGTGTGCTCTTCCGATCT
