# Supplementary material for: A broad analysis of splicing regulation in yeast using a large library of synthetic introns
Source: PLoS Genet. 2021 Sep 27;17(9):e1009805. doi: 10.1371/journal.pgen.1009805 (PMC8496845; doi:10.1371/journal.pgen.1009805)
Supplement: S3 Data — (PDF) [file pgen.1009805.s011.pdf]

Consensus Alignment: S. cerevisiae MUD1, modified MUD1, Untitl...

GAAACAAAAAAAAAAAAATGGAAGTCAGGGTGTTGGTGTAAAGCTGCTATCAACAAAAGACGGCCTCAGGCACTCAAACCTAAAGAAACCATGTCAGCG
consensus sequence Untitled Consensus

GAAACAAAAAAAAAAAAATGGAA----------------AAAGCTGCTATCAACAAAAGACGGCCTCA-----TCAAACCTAAAGAAACCATGTCAGCG
aligned sequence S. cerevisiae MUD1

GAAACAAAAAAAAAAAAAGTGGAAGTCAGGGTGTTGGTGTAAAGNNNNNNNNTACGTTAAACAATACGAGGCACTTACTCCGNNNNNNNNNNNNTCAGCA
Cloning & Seq homology | Index | Library specific primer | Barcode
modified_MUD1
aligned sequence modified MUD1

TATGTATATACCTTGTAATTTACGTTTCCTTAAATCTTCTTTCTACTAACGTTTTCATTATTCTATACTCTATGACCAATAAAAACAGACTGTACTTTCA
consensus sequence Untitled Consensus

TATGTATATACCTTGTAATTTACGTTTCCTTAAATCTTCTTTCTACTAACGTTTTCATTATTCTATACTCTATGACCAATAAAAACAGACTGTACTTTCA
aligned sequence S. cerevisiae MUD1

AAGTCATATACCTTGTAATTTACGTTTCCTTAAATCTTCTTTCTGTAGTTGTTTTCATTATTCTATACTCTATGACCAATAAAAACGGACTGTACTTTCA
Library variable region
modified_MUD1
aligned sequence modified MUD1

AAATTTACCCAGTAGGCCAGCAAATAAAGAAAATTATACCAGATTACTTCTGAAACACATTAATCCCAACAACAAGTATGCCATTAATCCGTCGCTACCC
consensus sequence Untitled Consensus

AAATTTACCCAGTAGGCCAGCAAATAAAGAAAATTATACCAGATTACTTCTGAAACACATTAATCCCAACAACAAGTATGCCATTAATCCGTCGCTACCC
aligned sequence S. cerevisiae MUD1

AAATTTACCCAGTAGGCCAGCAAATAAAGAAAATTATACCAGATTACTTCTGCCTGGAGTTCGCTATTCCTATTGTAGTTTTTCAATGTGACTGCGTTCC
Library variable region | Library specific primer | Cloning & ... homology
modified_MUD1
aligned sequence modified MUD1

CTTCCCCACAATAAATTACAAATATCATCACAACCGCTGATGTTACTAGATGATCAAATGGGCCTTCTTGAAGTTTCTATTTCAAGATCATCAAAGATGA
consensus sequence Untitled Consensus

CTTCCCCACAATAAATTACAAATATCATCACAACCGCTGATGTTACTAGATGATCAAATGGGCCTTCTTGAAGTTTCTATTTCAAGATCATCAAAGATGA
aligned sequence S. cerevisiae MUD1

ACTCCCCACAATAAATTACAAATATCATCACACCCGCTGATCTTACTAGGTGATCAACTGGGCCTTCTTGAAGTTTCTATTTCAAGATCATCAAAGCTGA
modified_MUD1
aligned sequence modified MUD1

CTAACCAAGCATTTTTAACGTTTGTCACTCAAGAAGAAGCAGACCGGTTTCTAGAAAAATACACGACAACAGCATTAAAAGTTCAAGGTCGTAAAGTGAG
consensus sequence Untitled Consensus

CTAACCAAGCATTTTTAACGTTTGTCACTCAAGAAGAAGCAGACCGGTTTCTAGAAAAATACACGACAACAGCATTAAAAGTTCAAGGTCGTAAAGTGAG
aligned sequence S. cerevisiae MUD1

CTAACCAAGCATTTTTAACGTTTGTCACTCAAGAAGAAGCAGACCGGTTTCTAGAAAAATACACGACAACAGCATTAAAAGTTCAAGGTCGTAAAGTGAG
modified_MUD1
aligned sequence modified MUD1

AATGGGAAAGGCTCGAACAAATTCGTTATTGGGTCTTTCAATAGAAATGCAAAAAAAAAAAGGTAATGACGAAACGTACAACCTTGATATAAAGAAGGTG
consensus sequence Untitled Consensus

AATGGGAAAGGCTCGAACAAATTCGTTATTGGGTCTTTCAATAGAAATGCAAAAAAAAAAAGGTAATGACGAAACGTACAACCTTGATATAAAGAAGGTG
aligned sequence S. cerevisiae MUD1

ACTGGGAAAGACTCGAACAAATTCGTTATTGGGTCATTCAATAGAAATCAAAAAAAAAAAAGGTAATTACGAAACGTACAACCTTGATATAAAGAAGGTG
modified_MUD1
aligned sequence modified MUD1

CTTAAAGCAAGGAAACTTAAACGCAAGTTACGTAGTGATGATATATGCGCCAAAAAGTTCAGGCTTAAAAGGCAAATAAGACGCCTGAAGCATAAGCTGA
consensus sequence Untitled Consensus

CTTAAAGCAAGGAAACTTAAACGCAAGTTACGTAGTGATGATATATGCGCCAAAAAGTTCAGGCTTAAAAGGCAAATAAGACGCCTGAAGCATAAGCTGA
aligned sequence S. cerevisiae MUD1

CTTAAAGCAAGGAAACTTAAACGCAAGTTACGTAGTGGTGATATTTGCGCCACAAAGTTCAGGCTTAAAAGGCAAATAAGACGCGTGAAGCATAAGCTGA
modified_MUD1
aligned sequence modified MUD1

GATCAAGAAAGGTAGAGGAGGCTGAGATTGATAGGATTGTAAAAGAATTTGAAACCCGTAGATTGGAAAACATGAAGTCTCAGCAAGAAAATCTAAAACA
consensus sequence Untitled Consensus

GATCAAGAAAGGTAGAGGAGGCTGAGATTGATAGGATTGTAAAAGAATTTGAAACCCGTAGATTGGAAAACATGAAGTCTCAGCAAGAAAATCTAAAACA
aligned sequence S. cerevisiae MUD1

GATCAAGAAAGGTAGAGGAGGCTGAGAGTGATAGGATTGTAAAAGAATTTGAAACCCGTAGATTCGAAAACATCAAGTCTCAACCAGAAAATCTAAAACA
modified_MUD1
aligned sequence modified MUD1

ATCGCAGAAACCTCTTAAGCGGGCTAAAGTGTCCAATACAATGGAAAATCCACCGAACAAAGTCCTTCTTATACAAAATTTGCCAAGCGGCACTACCGAG
consensus sequence Untitled Consensus

ATCGCAGAAACCTCTTAAGCGGGCTAAAGTGTCCAATACAATGGAAAATCCACCGAACAAAGTCCTTCTTATACAAAATTTGCCAAGCGGCACTACCGAG
aligned sequence S. cerevisiae MUD1

ATCGCAGAAACCTCTTAAGCGGGCTAAAGTGTCCAATACAAAGGAAAATACACCGAACAAGGTCCTTCTTATACAAAATTTGCCTAGCGGCACTACCGAG
modified_MUD1
aligned sequence modified MUD1

CAATTATTGTCGCAAATACTTGGCAATGAGGCTTTAGTTGAAATCAGATTAGTTAGCGTTCGTAACCTAGCTTTCGTGGAATACGAGACCGTTGCTGATG
consensus sequence Untitled Consensus

CAATTATTGTCGCAAATACTTGGCAATGAGGCTTTAGTTGAAATCAGATTAGTTAGCGTTCGTAACCTAGCTTTCGTGGAATACGAGACCGTTGCTGATG
aligned sequence S. cerevisiae MUD1

CAATTATCCTCGCAAATACTTGGCAAGGAGGTTTTAGTTGAAATCAGTTTAGTTAGCGTTCGTTACCTAGCTTTCGTGGAATACGAGACCGTTGCTGTTG
modified_MUD1
aligned sequence modified MUD1

CTACGAAAATCAAGAATCAGTTAGGCTCCACTTACAAGCTACAAAACAATGACGTTACCATAGGATTTGCTAAGTAGAATTTCCTTTGCGGAAGTATACC
consensus sequence Untitled Consensus

CTACGAAAATCAAGAATCAGTTAGGCTCCACTTACAAGCTACAAAACAATGACGTTACCATAGGATTTGCTAAGTAGAATTTCCTTTGCGGAAGTATACC
aligned sequence S. cerevisiae MUD1

CTACGAAAATCAAGAATCAGTTAGGCTCCACTTACAAGCTACAAAACAATAACGTTACCATAGGATTTGCTAAGTAGAATATCCTTTGCGGAAGTATACC
modified_MUD1
aligned sequence modified MUD1

TCGAGTAAAGAAATTCACAGATAAATTTGAATAACGTTCTCCATTATGAATAAATATGTATTTACAAGCTTAGAAAATAATGTGCTCTTTATTTACC
consensus sequence Untitled Consensus

TCGAGTAAAGAAATTCACAGATAAATTTGAATAACGTTCTCCATTATGAATAAATATGTATTTACAAGCTTAGAAAATAATGTGCTCTTTATTTACC
aligned sequence S. cerevisiae MUD1

TCGAGTAAAGAAATTCACAGATAAATTTGAATAACGTTCTCCATTATTAATAAATATTTATTTACAAGCTTAGAAAATAAGGTGCTCTTTATTTACC
modified_MUD1
aligned sequence modified MUD1
