# Supplementary material for: A broad analysis of splicing regulation in yeast using a large library of synthetic introns
Source: PLoS Genet. 2021 Sep 27;17(9):e1009805. doi: 10.1371/journal.pgen.1009805 (PMC8496845; doi:10.1371/journal.pgen.1009805)
Supplement: S4 Data — (PDF) [file pgen.1009805.s012.pdf]

1. Gradient boosting model predictions – contingency table

predicted \ measured	Unspliced [$SE \leq 0.05$]	Intermediate splicing [$0.05 < SE < 0.75$]	High splicing [$SE \geq 0.75$]
Unspliced [$SE \leq 0.05$]	47.3%	1%	0.06%
Intermediate splicing [$0.05 < SE < 0.75$]	23.9%	15.3%	9.5%
High splicing [$SE \geq 0.75$]	0.3%	0.6%	2%

$$\varphi_C = 0.191$$

2. Multiple barcodes pairwise comparison – contingency table

BC 2 \ BC 1	Unspliced [$SE \leq 0.05$]	Intermediate splicing [$0.05 < SE < 0.75$]	High splicing [$SE \geq 0.75$]
Unspliced [$SE \leq 0.05$]	29.3%	7.6%	0.5%
Intermediate splicing [$0.05 < SE < 0.75$]	7.6%	11.3%	9.9%
High splicing [$SE \geq 0.75$]	2.7%	5%	25.7%

$$\varphi_C = 0.279$$

BC 3 \ BC 1	Unspliced [$SE \leq 0.05$]	Intermediate splicing [$0.05 < SE < 0.75$]	High splicing [$SE \geq 0.75$]
Unspliced [$SE \leq 0.05$]	33%	9.7%	0.9%
Intermediate splicing [$0.05 < SE < 0.75$]	9.7%	10.7%	4.9%
High splicing [$SE \geq 0.75$]	1.9%	5.8%	22.3%

$$\varphi_C = 0.28$$

BC 4 \ BC 1	Unspliced [$SE \leq 0.05$]	Intermediate splicing [$0.05 < SE < 0.75$]	High splicing [$SE \geq 0.75$]
Unspliced [$SE \leq 0.05$]	34.1%	5.9%	1.2%
Intermediate splicing [$0.05 < SE < 0.75$]	9.4%	14.1%	4.7%
High splicing [$SE \geq 0.75$]	2.4%	5.9%	21.2%

$$\varphi_C = 0.313$$

BC 3 \ BC 2	Unspliced [$SE \leq 0.05$]	Intermediate splicing [$0.05 < SE < 0.75$]	High splicing [$SE \geq 0.75$]
Unspliced [$SE \leq 0.05$]	31.1%	11.7%	1%
Intermediate splicing [$0.05 < SE < 0.75$]	10.7%	8.7%	6.8%
High splicing [$SE \geq 0.75$]	1%	6.8%	20.4%

$$\varphi_C = 0.24$$

BC 4 \ BC 2	Unspliced [$SE \leq 0.05$]	Intermediate splicing [$0.05 < SE < 0.75$]	High splicing [$SE \geq 0.75$]
Unspliced [$SE \leq 0.05$]	35.3%	7%	0%
Intermediate splicing [$0.05 < SE < 0.75$]	7.1%	14.1%	4.7%
High splicing [$SE \geq 0.75$]	1.2%	4.7%	22.4%

$$\varphi_C = 0.376$$

BC 4 \ BC 3	Unspliced [$SE \leq 0.05$]	Intermediate splicing [$0.05 < SE < 0.75$]	High splicing [$SE \geq 0.75$]
Unspliced [$SE \leq 0.05$]	32.9%	10.6%	0%
Intermediate splicing [$0.05 < SE < 0.75$]	11.8%	10.6%	7.1%
High splicing [$SE \geq 0.75$]	0%	4.7%	20%

$$\varphi_C = 0.295$$
