# Supplementary material for: A broad analysis of splicing regulation in yeast using a large library of synthetic introns
Source: PLoS Genet. 2021 Sep 27;17(9):e1009805. doi: 10.1371/journal.pgen.1009805 (PMC8496845; doi:10.1371/journal.pgen.1009805)
Supplement: Attachment — Submitted filename: PLOS Genetics response to reviewers.pdf [file pgen.1009805.s014.pdf]

מחלקה לגנטיקה מולקולרית

Department of Molecular Genetics

מכון ויצמן למדע

רחובות 76100

טלפון 08-9343970

פקס 08-9344108

Weizmann Institute of Science

76100 Rehovot, Israel

Phone 972-8-9343970

Fax 972-8-9344108

Tuesday, August 17, 2021

Professor Yitzhak Pilpel
Head, Department of Molecular Genetics
Head, Azrieli Institute of Systems Biology
Weizmann Institute of Science
Email Pilpel@weizmann.ac.il
http://longitude.weizmann.ac.il/

Re: Revision on PGENETICS-D-21-00763

Dear Dr. Ast, PLoS Genetics editors,

We thank you for considering our manuscript, titled “A broad analysis of splicing regulation in yeast using a large library of synthetic introns”, for publication in PLOS Genetics and we wish to thank the referees for their detailed and thorough review reports. These comments helped us to improve our manuscript, and we hope you will find our revised manuscript fit for publication in PLOS Genetics.

Please find below a detailed point-by-point response to the referees’ comments marked in blue.

Sincerely,
Yitzhak (Tzachi) Pilpel
Tzachi Pilpel

Reviewer 1:

Schirman et al took a synthetic approach to study cis regulatory elements of alternative splicing. Many of their finding as the importance of the 3' and 5' SS and BS are known, but their findings might be able to fine tune our understanding of these seq.

Schirman's results find that the most important feature is the sequence of the BS and that its importance is associated with the presence or absence of U2AF1 which binds this seq. In addition the length between the BS-to-3'SS is also of importance.

Using AI Schirman can predict 70% of splicing efficiency. Interestingly 30% of splicing events were affected by the barcode seq that was in the exon.

The manuscript is easy to read and the figures are well presented.

We thank the reviewer for appreciating the contribution of our manuscript for our understanding of constitutive and alternative splicing regulation.

Major points:

1. I find that the most interesting finding is that splicing efficiency is positively correlated with RNA abundance. This result should be further explored. All the library has the same promoter but does transcription initiation is indeed similar? How about elongation rate? Can the sequence change the elongation rate? Maybe the authors can use their RNAseq data to explore these options.

   We thank the reviewer for this thoughtful comment. According to the literature, we believe that the most probable mechanism contributing to this correlation would be

related to degradation of unspliced mRNA through the NMD pathway (Sayani et al 2008 PMID: 18691968, Celik et al 2017 PMID: 28209632), or through inefficient nuclear export of unspliced mRNA (Zhou et al 2000 PMID: 11014198), as we now make clearer in the manuscript (Page 12, lines 258-262, and page 30 lines 671-686). In addition, we agree with the reviewer that sequence variation in our library might affect transcription elongation rate. We believe our dataset of steady state RNAseq is not suitable for measuring changes in transcription elongation rate within the library. In order to truly measure changes in transcription elongation rate, one must use a method to follow RNA Pol II dynamics using nascent RNA sequencing (possibly NET-seq (Churchman and Weissman 2011 PMID: 21248844)) which would be challenging to adapt for a pooled library and we believe is out of the scope of this project.

Nevertheless, we can apply bioinformatic tools on our library's sequences to gain some insights on possible effects of library variants on RNA abundance , e.g. through effects on transcription elongation. We applied a tool for nucleosome occupancy prediction (Xi et al 2010, PMID: 20576140) for each variant of the library. The library variable region resides at the start of the transcript where the first nucleosome is expected to reside, and we found that fully spliced variant are mostly predicted not to have a nucleosome at their 5' end compared to unspliced, and intermediate splicing groups (See Figure 1 below). Yet, since nucleosome occupancy is known to be correlated with GC content we controlled for GC content by randomly choosing variants with the same GC content distribution as the fully spliced group, and the observation still holds. As the peak of nucleosome occupancy at transcripts' 5' end was shown to be negatively correlated with transcription elongation efficiency (Choi et al 2009 PMID: 19723310) this analysis lends some support for the hypothesis of that the correlation between splicing

efficiency and RNA abundance in our library could in part be mediated through transcription elongation. However, we note that this analysis is mostly speculative and is based only on a computational prediction, hence we chose not to include it in our manuscript.

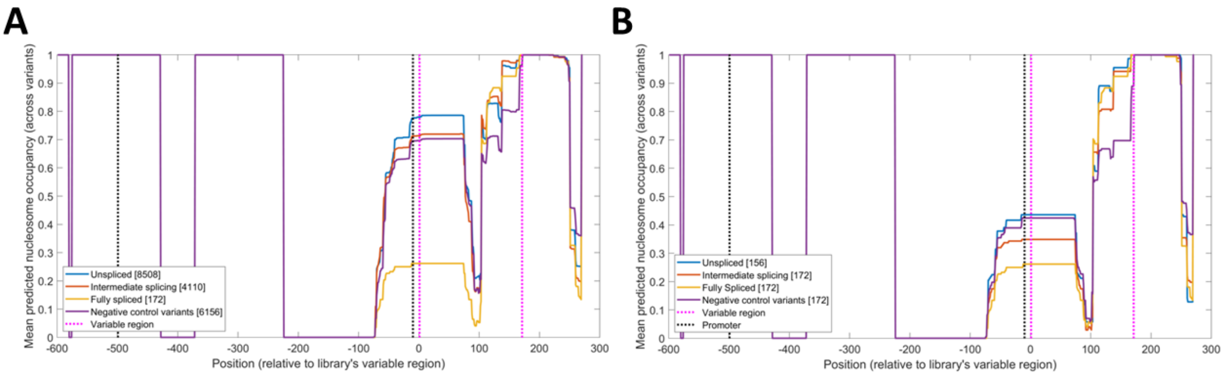

Figure 1 - Fully spliced variants are predicted to be less occupied by nucleosome
A. Averaged predicted nucleosome occupancy for four different groups of variants along the library's transcript. The blue line represents the group of unspliced variants (splicing efficiency = 0), the yellow line represents the group of fully spliced variants (splicing efficiency = 1), the red line represents all variants with intermediate splicing efficiency values, and the purple line represent the group of negative control variants. Promoter region is marked between two dashed black lines, the library's variable region is marked between two pink dashed lines. **B.** Same as (A), but here all the groups were randomly sampled from the same GC content distribution as the fully spliced group.

2. The connection of BS and U2AF1 can be tested by inserting U2AF1 to cerevisiae and validating the result with a couple of transcripts.

We thank the reviewer for this suggestion, we agree that such an experiment can contribute greatly to establishing the role of U2AF1 in the yeast splicing machinery, and we gave a lot of thought for doing it. However, we believe that accomplishing such an advance will require many trials and errors and a lot of calibrations, beyond the current scope. So unfortunately we would rather not do this major additional project at this stage.

The reason for the difficulty is that U2AF1, acts as a hetero-dimer with U2AF2. Using multiple sequence alignment of *S. cerevisiae* U2AF2 ortholog (*MUD2*), with U2AF2 sequences of the yeast strains that have U2AF1 we found that U2AF2 is poorly conserved between *S. cerevisiae* and these species (see Figure 2 below). Hence, we believe that merely expressing U2AF1 from one of these yeast species might not result with it interacting with the *S. cerevisiae* version of U2AF2 to create a hetero-dimer and therefore will not incorporate into the splicing machinery. A proper, but much broader undertaking may require the simultaneous incorporation of multiple genes.

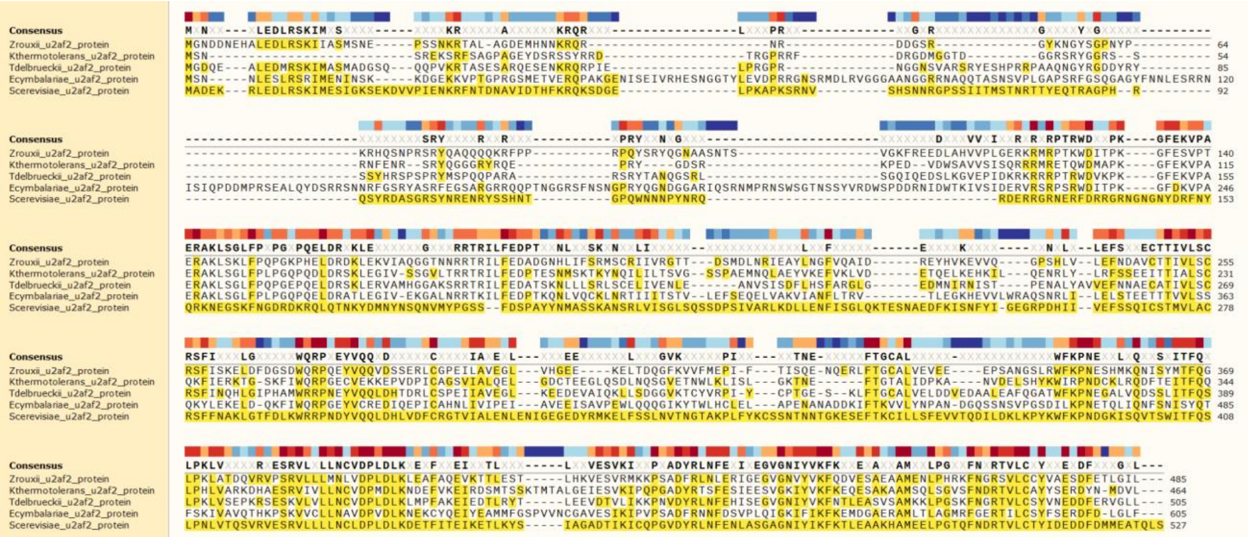

Figure 2 - U2AF2 multiple sequence alignment

Multiple sequence alignment of *S. cerevisiae*'s *MUD2* (U2AF2 ortholog) with U2AF2 of the four yeast species represented in our manuscript that have a copy of U2AF1 (*Z. rouxii, K. thermotolerans, E. cymbalariae, T. delbrueckii*)

Reviewer 2:

Schirman, et al. analyze the determinants of constitutive splicing and alternative splicing in yeast using a library of intron sequence variants that span a range of design strategies including both synthetic and natural introns. The splicing measurements for this library corroborate known components of splicing regulation including consensus splice sites, distance requirements for 3'

splice site selection, and dependence on GC content. They also uncover new determinants for steady state splicing levels and cryptic splicing, including the preference for a poly-U tract and the presence of a non-canonical 3'SS. The authors identify patterns in S. cerevisiae splicing of orthologous introns and explore the capacity to generate two-intron genes that are alternatively spliced.

The description of the library design and results are clear, and the insights derived from analysis of their library merit publication in PLOS Genetics. The splicing efficiency values provided in the supplement will serve as a powerful resource for future analyses of determinants of splicing in yeast. We ask that the authors respond to the following minor suggestions and requests for clarification.

We thank the reviewer for their appreciation of our manuscript, and its contribution for the body of knowledge on splicing in yeast.

<u>Minor comments:</u>

1. A couple questions on the data quality: What can be said about the correlation between replicates for intermediate values of splicing efficiency, excluding constitutively spliced introns (splicing efficiency ~1) along with introns that are not spliced at all (splicing efficiency ~0)? From Fig. S1, it appears that intermediate values are uncorrelated between replicates with different barcode sequences. Also, are any sequence coverage cutoffs for total RNA levels made before including a library variant in the analysis pipeline? Coverage cutoffs may improve the quality of splicing efficiency readouts.

   We agree that intermediate splicing values are not correlated between identical designs with different barcodes. We think that these differences reflect an effect of exonic sequences on splicing, however since the library was designed to study intronic regulatory sequence, we lack sufficient statistical power to gain insight on the effect of exonic sequences using this library.

Nevertheless, following this comment we did try to examine possible effects of the barcodes' sequence on splicing efficiency. We first counted the presence of Exonic Splicing Enhancers (ESE) in exon1 (where the barcode resides) for each variant. ESE motifs were taken from (Savisaar and Hurst 2016 PMID: 26802218). Figure 3 presents the same scatter plots as figure S1C from the manuscript, where the ESE count in exon1 for the variant with barcode x (where x denotes the row number in the figure) is depicted through a color code. If ESE levels had an effect on splicing efficiency we would expect to see enrichment of colored dots below the diagonal. In Figure 4 we repeated the same analysis for predicted RNA secondary structure at a 30 nt window around the 5'SS, since the barcode sequence might base pair with the 5'SS and affect its structure. In this analysis as well, if differences in secondary structure would have explained differences between barcodes we would expect to see enrichment in higher dG values below the diagonal. For both analyses we did not observe any significant contribution of the feature of interest. Using these two analyses we rule out contribution of ESE motifs and predicted RNA secondary structure to the barcode effect, but we still think that it is probable that 12 nt upstream exonic sequence has an effect on splicing efficiency which cannot be quantitatively measured using this library.

We used a read cutoff value of 10 reads per variant (i.e. variants with <10 reads were discarded).This information is now reported in page 40 line 961.

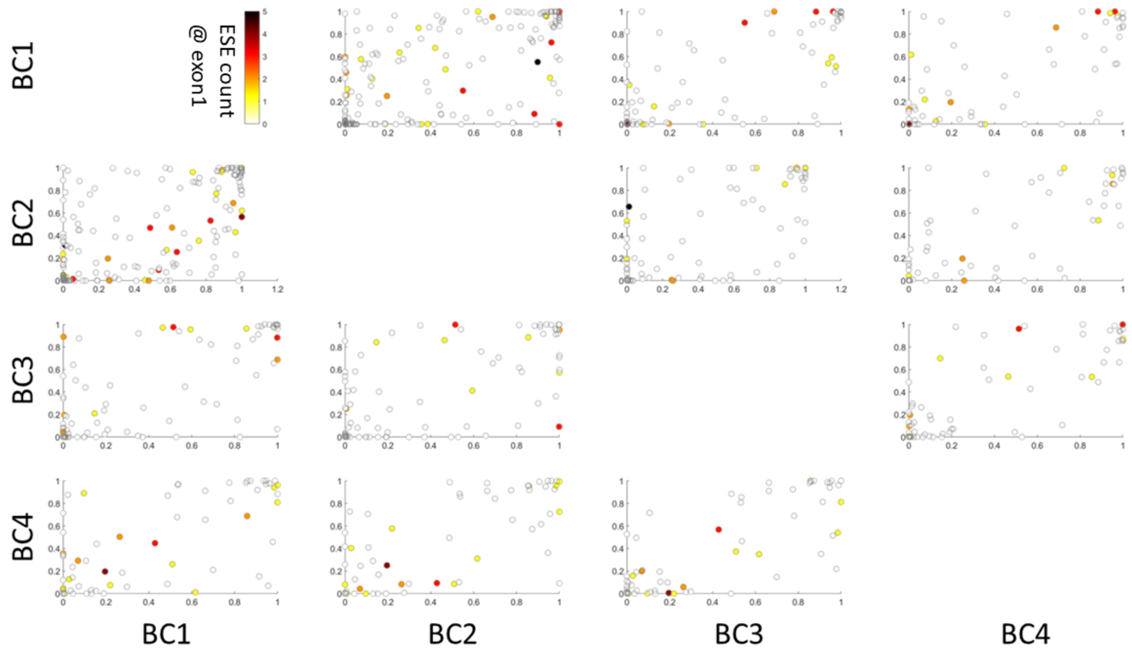

Figure 3 - Variability between identical designs with different barcodes is not explained by ESE motifs.

Similar to figure S1C in the manuscript, colors denote the number of ESE motifs (as taken from Savisaar and Hurst 2016) in exon1 for the variant with barcode number corresponding to the row number.

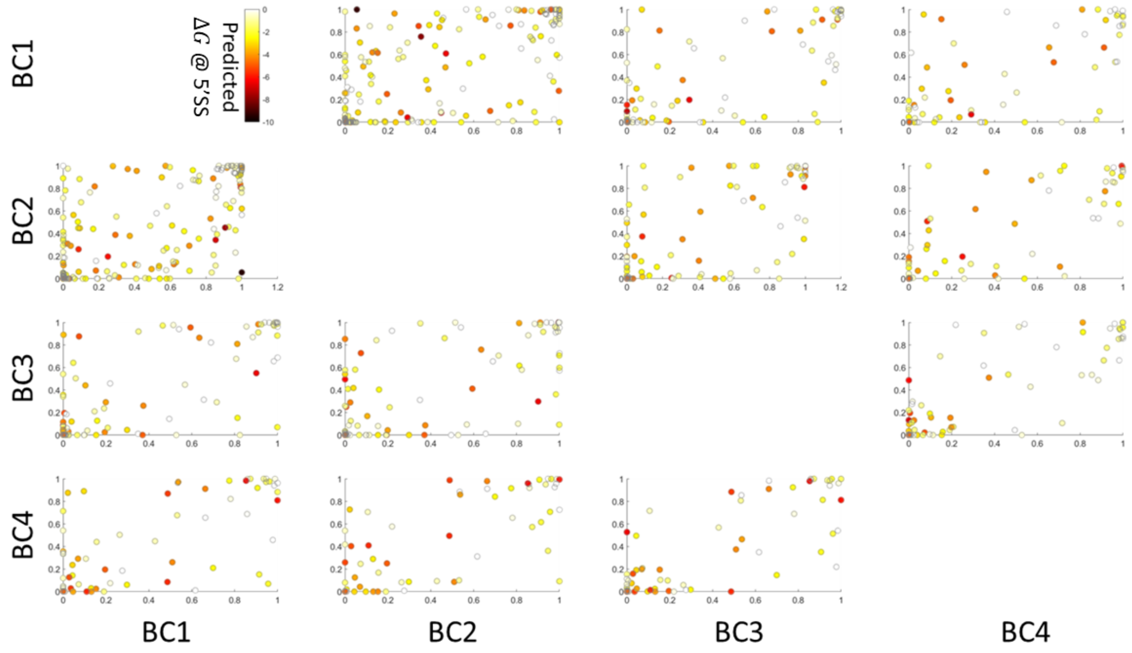

Figure 4 - Variability between identical designs with different barcodes is not explained by RNA secondary structure at 5' splice site

Similar to figure S1C in the manuscript, colors denote the RNA structure free energy (dG) of a region of 30nt centered at the 5'SS as predicted by Vienna RNAfold for the variant with barcode number corresponding to the row number.

2. Given that the Pearson correlation between replicates from Fig. S1 is ~0.76, it is surprising that the gradient boosting model was able to reach a Pearson correlation of ~0.77 between predictions and the experimentally observed splicing efficiency values. How are the data split between the 75% training/validation set and 25% test set? Are highly similar library variants present in both training/validation and test sets? It would be good to check that the model is able to make predictions on intron variants sufficiently different from examples in the training set.

   We agree that the Pearson correlation of the model predictions is similar to the Pearson correlation between identical designs with multiple barcodes, and that one might expect that model predictions will typically present lower correlation with measured signal compared to correlations between repeats. However we wish to note that in the case of agreement between different barcodes we observe many cases of same splicing efficiency values when both barcodes result in fully spliced intron or fully unspliced intron, and as noted by the reviewer the variance between the repeats is due to ~33% of the variants with intermediate splicing efficiency values. On the other hand, for the model predictions we observe a more continuous scale of predicted splicing efficiency values and the correlation is observed throughout the range. Therefore, we suggest that Pearson correlation is not the right statistical measure to compare between the two sets. Instead we compare between the two sets using a contingency table with three classes [unspliced, intermediate splicing, fully spliced], and use Cramer's V value ($\boldsymbol{\varphi}_C$) for each table as an effect size metric. We found that the effect size for the comparison between two specific barcodes sets is $\boldsymbol{\varphi}_C$=0.278 and the average for all pairwise comparisons

between barcodes sets is φ_C=0.297, while the comparison between model predictions and measured values for the test set yields a lower agreement of φ_C=0.191. We have now revised accordingly the section in the manuscript that conveys the model prediction accuracy to better reflect this nature of the correlation (page 25, lines 550-565).

As for the other comments, the variants were split randomly between a training set and a test set. As the test set is composed of 25% of the variants we can be sure it includes all single intron design types from our library.

We tried to run the model again, this time assigning a specific group of designs for the test set. We assigned 2,437 variants of natural introns (including *S. cerevisiae* introns, ortholog introns, and mutated *S. cerevisiae* introns) as the test set, and used 10,230 synthetic variants as training set. In this case we got a Pearson correlation of r=0.87 and Cramer's V of φ_C=0.29. But since the training/test split here is not random we don't think this analysis is more suitable.

Contingency table 1 – multiple barcodes

BC 1 / BC 2	Unspliced [$SE \leq 0.05$]	Intermediate splicing [$0.05 < SE < 0.75$]	High splicing [$SE \geq 0.75$]
Unspliced [$SE \leq 0.05$]	29.3%	7.6%	0.5%
Intermediate splicing [$0.05 < SE < 0.75$]	7.6%	11.3%	9.9%
High splicing [$SE \geq 0.75$]	2.7%	5%	25.7%

$$\varphi_C = 0.279$$

Contingency table 2 – gradient boosting model

measured / predicted	Unspliced [$SE \leq 0.05$]	Intermediate splicing [$0.05 < SE < 0.75$]	High splicing [$SE \geq 0.75$]
Unspliced [$SE \leq 0.05$]	47.3%	1%	0.06%
Intermediate splicing [$0.05 < SE < 0.75$]	23.9%	15.3%	9.5%
High splicing [$SE \geq 0.75$]	0.3%	0.6%	2%

$$\varphi_C = 0.191$$

3. A component of the library included introns with a different background sequence, but these were not discussed further. How much does background sequence effect splicing

efficiency levels? This information could inform the generalizability of studies that use mini-gene constructs based on single background sequences.

We thank the reviewer for this comment. In the revised manuscript we added this analysis as a supplementary figure (Figure S3A), and refer to it in the text (page 15, lines 332-343). In short we now note in the manuscript that the background sequence can have a significant effect either increasing or decreasing splicing efficiencies. On average the two background sequences coming from intron-containing genes from *S. cerevisiae* result in more efficient splicing than random sequences.

4. The authors point out a paradox -- the 3'SS sequence AAG is suppressed in natural sequences compared to YAG, but their results show that AAG is as efficient as YYG in their synthetic sequences. A reconciliation suggested by authors is that AAG results in more cryptic events than YAG in their synthetic introns and so might be selected against in nature. However, there is also earlier work explaining the preference for pyrimidines at the -3 position based on structural interactions (e.g., the P state spliceosome structure, Wilkinson, et al. Science 2017). So why then is it that AAG splicing is as efficient as YAG splicing for the synthetic introns in the library? Could it be that these synthetic introns have other qualities (e.g. irregular lengths, non-consensus 5'SS / BP) that are the more relevant bottlenecks for splicing efficiency?

   This is certainly a point of interest. The mentioned reference by Wilkinson, *et al*. presents a cryo-EM structural analysis of the 3' splice site recognition by the *S. cerevisiae* spliceosome in its P-complex formation. This paper presents the structural basis for interaction between the spliceosome and a -3 pyrimidine at the 3' splice site based on a single intron of interest with an UAG 3'SS, which provides an explanation for the majority of natural splice sites being YAG. However, we don't think this result contradicts our results, since the previous work did not measure the structure with an intron utilizing AAG 3'SS, prohibiting a proper comparison.

We agree that our results regarding efficient splicing of introns utilizing AAG 3'SS are surprising, both in light of structural analysis and 3' splice site abundance in the genome. We thus believe our results here provide a challenge that may be studied further with structural analysis of an intron with AAG 3'SS.

We now refer to the important point regarding the cryo-EM P-complex structure in the revised manuscript (page 14, line 314-316).

As for other confounding factors that might affect our results regarding AAG 3'SS, we checked whether distribution of other features differ between variants with the 3 different splice sites and we did not detect any confounding factor. In addition, we used our gradient boosting model on a set of only introns with AAG 3'SS and compared the results of the feature importance analysis to a similar model for UAG or CAG 3'SS variants and we did not observe any significant difference in the inferred feature importance.

5. Why is it that intron lengths of 30-40 nucleotides are highly disfavored compared to >40 nucleotides (Fig. 3B)?

   We thank the reviewer for this comment. Following this comment we reexamined this analysis and discovered that the 30-40 bin included a large set of variants with mutations aimed to create stem-loop structures at the splice sites location. All these variants were designed with a BS-to-3'SS distance of 30 nt, and, as can be seen in Figure S2D of the revised manuscript, these mutations reduce appreciably the splicing efficiency.

   Thanks to this comment we removed this set of variants from this analysis. The revised figure can be seen in Figure 3B of the revised manuscript, and it now presents a negative correlation between BS-to-3'SS distance and splicing efficiency for distances longer than 20nt.

6. A note about the U2AF1 conclusions – the species with U2AF1 are also more evolutionarily distant from *S. cerevisiae*, so findings that the intron BP-3'SS length

distributions are different in those species may not be a result of only presence vs absence of U2AF1.

We note that *K. lactis* is also phylogenetically distant from *S. cerevisiae* at least as much as the four species with U2AF1 are, however it also lacks U2AF1 (Figure 5A). Nonetheless, its BS-to-3'SS distribution (Figure S4A) and its introns' splicing efficiency values distribution in our library (Figure 5A,B) are similar to *S. cerevisiae* and the other species without U2AF1. We believe that this case gives better support to our hypothesis regarding the presence/absence of U2AF1 compared to merely phylogenetic distance. We added a sentence in the manuscript referring to *K. lactis* phylogenetic distance (page 22, lines 487-489)

7. For Fig. 2A what are the units for total RNA abundance? Is this a log scale (base 2)?

   We thank the reviewer for this comment and revised the figure's label to include the units (log10 ratio between RNA and DNA normalized reads).

8. The authors mention that predicted secondary structure only had borderline effects on splicing efficiency. Can the authors include a description of the secondary structure analyses they conducted (perhaps in the supplement)? Also, it could be useful to note that in wildtype introns in *S. cerevisiae*, the longer introns in ribosomal protein coding genes are the ones with predicted structures, and introns of these lengths are not included in this work.

   We thank the reviewer for their interest in this result and in the revised manuscript we included it as a supplementary figure (Figure S2D), and refer to it in the text (page 15, lines 323-331)

Reviewer 3:

Schirman and colleagues report a very detailed study of the sequence determinants of splicing in the yeast S. cerevisiae, performed using a large library of synthetic variants of a model intron.

Although previous studies have addressed similar questions using related methods, this work is distinguished by its scale and by the careful experimental design that allows the authors to quantify the effects of various parameters, such as the sequence of 5'SS, BS, 3'SS, and the length and nucleotide composition of introns. A particularly interesting finding concerns the splicing efficiency of orthologous introns, and its relation to the presence and absence of the U2AF1 homologs across yeast species.

We thank the reviewer for their appreciation of our work and the novel contribution of this large scale library.

Minor comments:

1. Although alternative splicing is uncommon in S. cerevisiae, some endogenous yeast introns are know to be spliced more efficiently/accurately than others (PMID 29254943). It would be interesting to see whether the high-throughput measurements shown here recapitulate previous measurements of splicing efficiency and accuracy in endogenous yeast genes.

   We thank the reviewer for directing us to this dataset of alternative splicing events in natural *S. cerevisiae* introns. As suggested, we compared all the alternative splicing events for WT *S. cerevisiae* from this paper that are manifested through alternative downstream 3'SS (as these are the types of events we observe in our library) to our data of alternative 3'SS selection (termed cryptic splicing in our manuscript) in natural *S. cerevisiae* introns. We do not observe significant correlation in splicing error frequency (SEF) after adapting our data's units to this paper's units (See Figure 5A below). Additionally we do not observe significant agreement in the identity of introns with alternative splicing events (see Figure 5B below).

   Yet, we must stress that we did not expect to find an agreement between the two datasets since alternative 3'SS selection is dependent on the sequence of the

downstream exon, and this sequence is completely different between the natural introns in their natural context as was measured in the referred paper, and in our library where all introns reside within the same synthetic context.

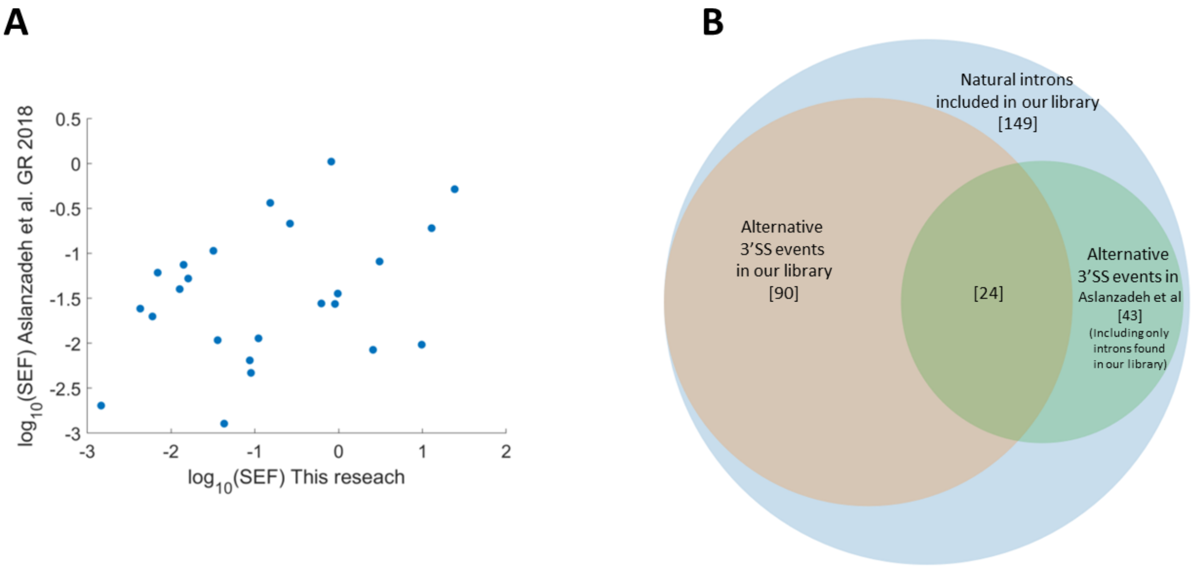

Figure 5 - Comparison of alternative splicing data for natural *S. cerevisiae* introns with Aslanzadeh, *et al*. 2018

A. Scatter plot of log_{10} of splicing error frequency (SEF) values (as defined in Aslanzadeh *et al*.) for natural *S. cerevisiae* introns for both datasets. Pearson correlation is not statistically significant (p-value = 0.08) **B.** Venn diagram depicting the intersection of introns with alternative splicing events in both datasets, the size of the intersection between the two datasets (24) is not larger than expected by chance (26).

2. There is a risk that some of the unspliced reads represent vector or genomic DNA contamination, rather than the intended cDNA molecules. How is this mitigated?

We agree that when analyzing pooled RNA samples there is a risk of genomic DNA contamination. Many times this risk is mitigated by sequencing constitutively spliced genes where you expect the RNAseq data to present only the spliced isoform and use that as a quality control. In this case the entire library is of an

intron-containing gene. Since we observe 486 variants in our library that are fully spliced, we can be sure that at least for these variants no DNA contamination was measured. Since all variants in the library are found with similar frequencies we believe we can safely assume that these 486 variants reflect on the entire library and that DNA contamination levels are negligible.

3. In Figure 1 (or S1), please include an alignment of the original MUD1 sequence and the synthetic construct sequence, indicating position coordinates, cloning sites, barcodes, and other relevant sequence elements. As it stands, the design is somewhat unclear, for example how can the 5' splice site be placed in position 6-11 of the oligo (Fig 1 legend), if the random barcode is placed in position 1-12 (Fig 1B)?

   We thank the reviewer for this comment, and in the revised manuscript we add the requested alignment as S11 - supplementary file 1. We hope that this helps to clarify the library design. In addition, following this comment we revised Figure 1 and the relevant text and now describe the 5'SS position as 18-23 when the barcode is at 1-12.

4. Figure 2C: For clarity, the labels should read "all consensus sites", and "1 or more non-consensus sites".

   We thank the reviewer for this comment and revised the figure labels as suggested.

5. Figure 3B shows a surprisingly large effect of BS-3'SS distance on splicing efficiency. For example, variants with 30-40 nt distance are almost never spliced, but other variants are spliced to various degrees. How confident are you that the effect is caused by distance per se, rather than some other sequence property (eg RNA folding or nucleotide composition)?

We thank the reviewer for this comment. Following this comment we reexamined this analysis and discovered that the 30-40 bin included a large set of variants with mutations aimed to create stem-loop structures at the splice sites location. All these variants were designed with a BS-to-3'SS distance of 30 nt, and as can be seen in Figure S2D of the revised manuscript these mutations highly reduce splicing efficiency.

Thanks to this comment we removed this set of variants from this analysis. The revised figure can be seen in Figure 3B of the revised manuscript, and it now presents a negative correlation between BS-to-3'SS distance and splicing efficiency for distances longer than 20nt.

6. Why were negative control designs excluded from the analysis in the computational model shown in Fig 7?

   Negative control variants in our library were created by replacing splice site sequences with random sequences. Therefore, we believe these variants will not even be recognized as intron by the splicing machinery. Indeed almost all of these variants in our dataset are unspliced.

   The main purpose of the computational model in this work was to infer the genetic features that are important for splicing using feature importance analysis. Inclusion of the negative control variants in the model would inflate the importance of the splice site sequence on the expanse of other features in a manner that we believe doesn't reflect the true biological properties of this system.

   As a demonstration of this, we run our model including the negative control variants and the difference in feature importance inference is demonstrated in Figure 6 below. One can notice that the importance of 5'SS and 3'SS sequences increased dramatically.

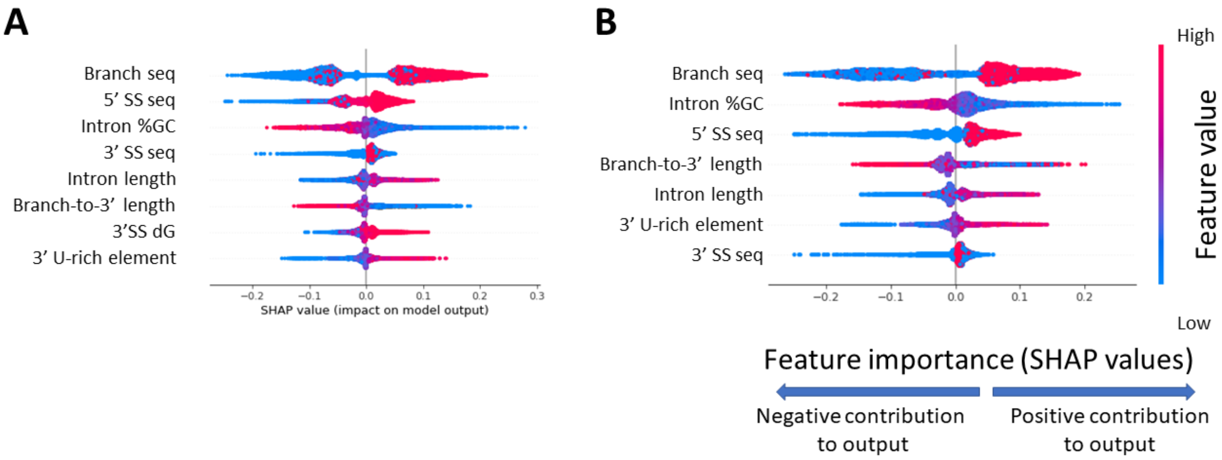

Figure 6 - Feature importance inference with and without negative control variants

A. Feature importance analysis based on SHAP values for the gradient boosting model including negative control variants. **B.** Feature importance analysis based on SHAP values for the gradient boosting model excluding negative control variants (identical to Figure 6B in the manuscript).
