# Supplementary material for: A broad analysis of splicing regulation in yeast using a large library of synthetic introns
Source: PLoS Genet. 2021 Sep 27;17(9):e1009805. doi: 10.1371/journal.pgen.1009805 (PMC8496845; doi:10.1371/journal.pgen.1009805)
Supplement: Attachment — Submitted filename: Schirman_etal_review.docx [file pgen.1009805.s013.docx]

**Review of “A broad analysis of splicing regulation in yeast using a large library of synthetic introns.”**

Schirman, et al. analyze the determinants of constitutive splicing and alternative splicing in yeast using a library of intron sequence variants that span a range of design strategies including both synthetic and natural introns. The splicing measurements for this library corroborate known components of splicing regulation including consensus splice sites, distance requirements for 3’ splice site selection, and dependence on GC content. They also uncover new determinants for steady state splicing levels and cryptic splicing, including the preference for a poly-U tract and the presence of a non-canonical 3’SS. The authors identify patterns in *S. cerevisiae* splicing of orthologous introns and explore the capacity to generate two-intron genes that are alternatively spliced.

The description of the library design and results are clear, and the insights derived from analysis of their library merit publication in PLOS Genetics. The splicing efficiency values provided in the supplement will serve as a powerful resource for future analyses of determinants of splicing in yeast. We ask that the authors respond to the following minor suggestions and requests for clarification.

- A couple questions on the data quality: What can be said about the correlation between replicates for intermediate values of splicing efficiency, excluding constitutively spliced introns (splicing efficiency ~1) along with introns that are not spliced at all (splicing efficiency ~0)? From Fig. S1, it appears that intermediate values are uncorrelated between replicates with different barcode sequences. Also, are any sequence coverage cutoffs for total RNA levels made before including a library variant in the analysis pipeline? Coverage cutoffs may improve the quality of splicing efficiency readouts.
- Given that the Pearson correlation between replicates from Fig. S1 is ~0.76, it is surprising that the gradient boosting model was able to reach a Pearson correlation of ~0.77 between predictions and the experimentally observed splicing efficiency values. How are the data split between the 75% training/validation set and 25% test set? Are highly similar library variants present in both training/validation and test sets? It would be good to check that the model is able to make predictions on intron variants sufficiently different from examples in the training set.
- A component of the library included introns with a different background sequence, but these were not discussed further. How much does background sequence effect splicing efficiency levels? This information could inform the generalizability of studies that use mini-gene constructs based on single background sequences.
- The authors point out a paradox -- the 3’SS sequence AAG is suppressed in natural sequences compared to YAG, but their results show that AAG is as efficient as YYG in their synthetic sequences. A reconciliation suggested by authors is that AAG results in more cryptic events than YAG in their synthetic introns and so might be selected against in nature.  However, there is also earlier work explaining the preference for pyrimidines at the -3 position based on structural interactions (e.g., the P state spliceosome structure, Wilkinson, et al. Science 2017). So why then is it that AAG splicing is as efficient as YAG splicing for the synthetic introns in the library? Could it be that these synthetic introns have other qualities (e.g. irregular lengths, non-consensus 5’SS / BP) that are the more relevant bottlenecks for splicing efficiency?
- Why is it that intron lengths of 30-40 nucleotides are highly disfavored compared to >40 nucleotides (Fig. 3B)?
- A note about the U2AF1 conclusions – the species with U2AF1 are also more evolutionarily distant from *S. cerevisiae*, so findings that the intron BP-3’SS length distributions are different in those species may not be a result of only presence vs absence of U2AF1.
- For Fig. 2A what are the units for total RNA abundance? Is this a log scale (base 2)?
- The authors mention that predicted secondary structure only had borderline effects on splicing efficiency. Can the authors include a description of the secondary structure analyses they conducted (perhaps in the supplement)? Also, it could be useful to note that in wildtype introns in *S. cerevisiae*, the longer introns in ribosomal protein coding genes are the ones with predicted structures, and introns of these lengths are not included in this work.
